# Report on the Forest Conditions of the Rocky Mountains

**Published:** 1889-08

**Authors:** 


REPORT ON THE FOREST CONDITIONS OF THE ROCKY MOUNTAINS and Other Papers; with a map showing the Location of Forest Areas on the Rocky Mountain Range, by B. E. Fernow, Chief of Forestry Division, Department of Agriculture, Washington, D. C., 1889. Bulletin No. 2.

In issuing this book, the Chief of the Forestry Division says:

"The following report and papers are designed as a basis for an intelligent conception of the possibilities and requirements of legislative action on the part of the General Government in regard to some of its property. They will also, it is hoped, be welcome to the student of the climatic, floral, and economic conditions of the region to which they refer, and serve as a historic reference book in the times when the folly of present days will be judged by those who will suffer its consequences."

The continued and even wanton destruction of timber in this country cannot be too severely and too frequently attacked nor too freely brought to the notice of people of all professions and trades, to the end that a great wave of public sentiment may arise that will stop the vandalism before it is too late. So great is this destruction that the Chief is provoked in this report to the savage but perfectly just remark: "That the beauty of the once verdant mountain sides is being ruthlessly and needlessly destroyed, and with such general equanimity is this devastation considered that we may soon substitute in our dictionaries the word 'Americanism' for 'Vandalism!'"

The report speaks of many of the beneficial effects of forests. Among them may be mentioned: Tendency to increase the water supply. They prevent the snows from melting too rapidly in the spring, and protect the springs and rivulets. They keep the ground moist and promote rain-fall. The headwaters of all important streams are protected and nourished by forests. Forests arrest the extreme desiccation of the atmosphere and rapid surface-drainage into the streams. Snow in the mountain forests melts more gradually than on bare surfaces, and thus forests prevent floods.

Considerable space is given in the report to the significant phenomena of avalanches and land-slides which are directly promoted by the cutting off of the trees. W. M. J.

